# Ag85A, As an S2 Vaccine Carrier, Reduces the Toxicity of the S2 Vaccine and Enhances the Protective Ability of Mice against Brucella

**DOI:** 10.1155/2022/4686541

**Published:** 2022-12-26

**Authors:** Shuai Zhang, Lumen Chao, Lemuge She, Humujile Sui, Huaxin Niu, Zeliang Chen, Xiangyang Li, Jingbo Zhai

**Affiliations:** ^1^Medical College, Inner Mongolia Minzu University, Tongliao 028000, China; ^2^College of Animal Science and Technology, Inner Mongolia Minzu University, Tongliao 028000, China; ^3^Key Laboratory of Zoonose Prevention and Control at Universities of Inner Mongolia Autonomous Region, Tongliao 028000, China; ^4^Brucellosis Prevention and Treatment Engineering Research Center of Inner Mongolia Autonomous Region, Tongliao 028000, China

## Abstract

Brucella is a globally distributed zoonotic disease that can cause abortion and changes in immune function in humans and animals. At present, there is no good treatment plan for Brucella, and animals can only be treated harmlessly once they become ill, resulting in huge economic losses. Therefore, the prevention of Brucella infection is a very crucial step. Although a variety of Brucella vaccines have been widely used, they have varying degrees of shortcomings. For example, some Brucella vaccines have residual virulence, which leads to the emergence of Brucella in animals during the immunization process. Bacillus infection and other conditions occur. To further reduce the toxicity of the Brucella vaccine and enhance its protective effect on animals, this study used Antigen 85A (Ag85A) as a carrier of the Brucella vaccine to fuse with the Brucella S2 vaccine. The results of the study found that the S2-Ag85A oral Brucella vaccine could effectively reduce the toxicity residue of the S2 vaccine, stimulate the mice to produce a better immunogenic response, and effectively activate the expression levels of Brucella heterozygous IgG1 and IgG2a. Experiments have shown that the expression of IFN-*γ* in the peripheral blood serum and spleen of mice is significantly increased, and the expression levels of IL-1*β*, TNF-*α*, and IL-6 are significantly reduced, which may indicate that S2-Ag85A oral Brucella vaccine could induce the expression of IFN-*γ*, thus downregulating the expression levels of IL-6 and TNF-*α* in the spleen tissue. The above results indicate that the S2-Ag85A oral vaccine is an effective attenuated vaccine for preventing Brucella infection.

## 1. Introduction

Brucella is a Gram-negative bacterium that can multiply in ruminants, and most mammals, causing abortion in pregnant animals, decreased immunity, infertility, and other problems, resulting in serious economic losses [1]. When humans are infected with Brucella, immune dysfunction, persistent high fever, spondylitis, arthritis, and infertility can occur; and in more severe cases, complications, such as meningitis and endocarditis, can occur [2–4]. Clinical symptoms of Brucella infection can last for more than one year and eventually evolve into a chronic attack.

At present, Brucella vaccines are widely used in animal husbandry, but most of the vaccines are attenuated vaccines. Attenuated vaccines have certain virulence residues while controlling Brucella infection. Attenuated Brucella vaccine can cause abortion in pregnant animals, and currently, there is a lack of a safe and effective Brucella vaccine [5, 6].

Antigen 85A (Ag85A) is one of the main members of the Ag85 protein family, and it is the main protein secreted outside the cell during the growth of Mycobacterium tuberculosis. Ag85A can potentially induce the level of Th1 cells and also can stimulate the quantity of CD4+ and CD8+ T cells to elicit an immune response [7]. Several studies demonstrated that Ag85A can influence the synthesis of single cells in the circulatory blood of infected cases who received vaccination with BCG and those with the combined disease, and it can improve immunogenicity in patients [8, 9].

Brucella S2-attenuated vaccine is widely used in livestock production, and its preventive effect is good. However, the S2 vaccine has similar shortcomings as most Brucella vaccines. As the S2 vaccine itself has a small amount of toxicity, people may develop brucellosis after its injection. Injection of the S2 vaccine in pregnant animals may cause abortion in pregnant livestock. In recent years, the safety of the S2 vaccine has been a research hotspot of Brucella vaccines [10–12].

The protection rate of the oral S2 vaccine against brucellosis is 40% to 60%; however, 20 billion units of oral S2 vaccine are provided now for immunizing the subjects [13–15], which ultimately increases the vaccine-related risk in animals. In order to increase the protective effect of the vaccine and reduce the side effects of excessive doses, we developed the oral-based S2-Ag85A DNA vaccine, using the antigen Ag85A to increase the immunogenicity in the body and to increase the titer of the vaccine.

## 2. Results

### 2.1. Construction of the S2-Ag85A Oral Vaccine

**Figure 1:**
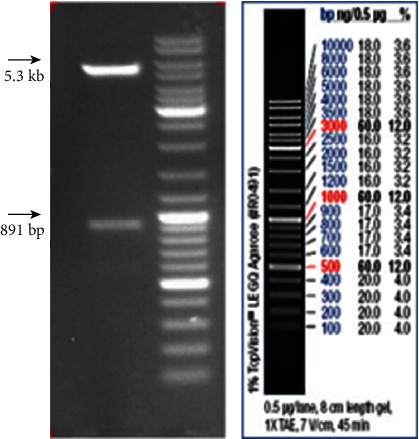
Recombinant Ag85A gel electrophoresis.

The S2-Ag85A oral Brucella vaccine was successfully constructed by the CRISPR/Cas9 technology and confirmed by polymerase chain reaction (PCR) and reverse transcription-polymerase chain reaction (RT-PCR). We used the primers in PCR and RT-PCR for amplifying the product of 891 bp, and no amplification product was obtained from the Ag85A mutant (Figure 1). These data indicate that the S2-Ag85A vaccine was constructed correctly.

The target band was 891 bp, and the carrier was 5.3 kb long.

### 2.2. Safety Evaluation of the S2-Ag85A Oral Vaccine

**Figure 2:**
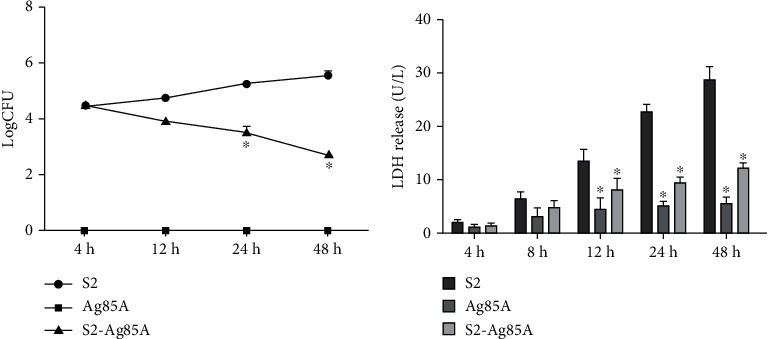
The S2-Ag85A oral vaccine can effectively inhibit the replication of Brucella in vitro. (a) The initial inoculation levels of S2, Ag85A, and S2-Ag85A were the same. After 12 hours of infection, the bacterial count was gradually increased in the S2 group from the baseline, and the bacterial count was gradually decreased in the S2-Ag85A. At 24 h and 48 h, the counted level of the virus was significantly decreased in the S2-Ag85A group than in the S2 group (*P* value < 0.05). (b) The releasing level of LDH in cells of each group at 4 h, 24 h, and 48 h was not significantly differentiated. There was a significantly lower level of LDH in the Ag85A group and S2-Ag85A group than that in the S2 group (*P* value < 0.05). ^∗^*P* value < 0.05 compared to the Ag85A group.

The main reason for the failure of the S2 vaccine is the small number of viruses carried in the S2 vaccine that replicate unrestrictedly in the body after being vaccinated, which not only plays a preventive role but also induces Brucella infection. The survival and replication of Brucella in macrophages are important pathogenic mechanisms of brucellosis. We evaluated the safety of the oral-based S2-Ag85A vaccine by inducing infection of RAW 264.7 macrophages with the S2 vaccine, oral-based S2-Ag85A vaccine, and Ag85A empty vector for comparing their growth capability at the intracellular level. Four hours after infection, the growth of Brucella in macrophages did not change significantly. At 12 h of infection, Brucella in the S2 vaccine group showed a growth trend, while Brucella in the S2-Ag85A group did not show any growth trend. At 48 h, the number of Brucella bacilli was increased in the macrophages from the Brucella S2 vaccine group, and a significantly decreased number of Brucella bacilli was found in the macrophages from the S2-Ag85A group (*P* < 0.05). This finding indicated that the oral-based S2-Ag85A vaccine was safe and stable in macrophages, without any risk of virus reproduction, and showed good safety (Figure 2(a)).

Furthermore, we tested the safety of the oral-based S2-Ag85A vaccine; we detected lactate dehydrogenase (LDH) secretion by macrophages and hemolytic activity. Our findings indicated that when the S2 vaccine, Ag85A vector and S2-Ag85aA oral vaccine acted on the macrophages, the secretion of LDH among the groups at 4 h was not significantly differentiated, and LDH secretion showed a significantly elevated level in the S2 group than that in the Ag85A vector group and the S2-Ag85A vector group at 12 h (*P* value < 0.05). With increasing the immunization time, the release of LDH was significantly elevated in the S2 group (*P* value < 0.05), and a significant level of LDH was increased than that in the Ag85A group and the S2-Ag85A group after S2 interacted with macrophages for 24 h and 48 h. After Ag85A interacted with macrophages for 12 h, the release of LDH was stable without an increasing trend. This finding showed that when Ag85A was used as the S2 vaccine carrier, it could effectively neutralize some of the toxicity induced by the S2 vaccine and reduce the secretion of LDH in macrophages (Figure 2(b)). The detection of hemolytic activity showed that there was no obvious hemolytic activity in each group after treatment for 0.25 h, and the hemolytic activity of the S2 group showed a significantly elevated level than that in the Ag85A group and S2-Ag85A group after 0.5-2 h of treatment (*P* value < 0.05). In the S2-Ag85A group, there was no hemolysis occurred (Table 1). The experimental results show that the Ag85A carrier can reduce the hemolysis caused by the S2 vaccine and enhance its safety.

### 2.3. The S2-Ag85A Oral Vaccine Attenuates Toxicity in Mice

**Figure 3:**
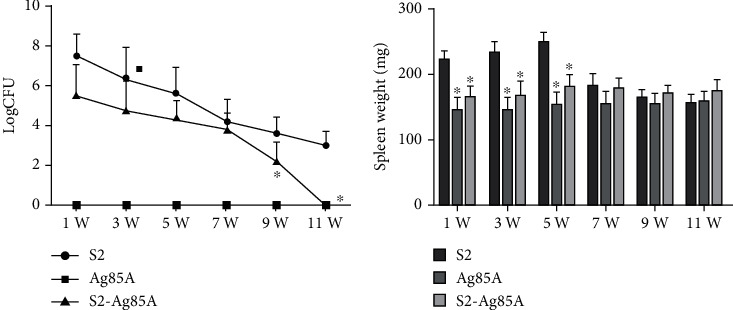
The S2-Ag85A oral vaccine attenuates toxicity in mice. (a) The bacterial content in the spleen was measured at the 1st, 3rd, 5th, 7th, 9th, and 11th week after the completion of vaccination, and the bacterial content showed significantly lower in the mice-spleen of the S2-Ag85A group than that in the S2 group at the 9th week and 11 weeks (*P* value < 0.05). (b) Determination of the spleen weight at the 1st, 3rd, 5th, 7th, 9th, and 11th weeks after the vaccination; the spleen of mice in the S2 group was swollen at the 1st, 3rd, and 5th week, and the weight elevated with a significant level (*P* < 0.05), and then gradually, it returned to normal; the similar weight of mice spleen was found in the S2-Ag85A and Ag85A groups. ^∗^*P* value < 0.05, compared with the S2 group.

After mice received the S2 vaccine, Ag85A vector, and S2-Ag85A vaccine by gavage, we measured the weight of the spleen and the number of Brucella bacilli in the spleen at 1, 3, 5, 7, 9, and 11 weeks after complete vaccination for evaluation. We found a significantly lower level of bacterial load in the spleen of mice vaccinated with S2-Ag85A (*P* < 0.05) at weeks 9 and 11 compared with mice in the S2 group, and it was completely cleared at week 11. The weight of the spleen in mice was measured, and it was found that the weight of mice receiving the S2-Ag85A oral vaccine was closer to the normal range at most time points; the S2 group showed early spleen enlargement, and the mouse weight was significantly higher than that after Ag85A and S2-Ag85A oral administration in the vaccine group (*P* < 0.05) (Figure 3).

### 2.4. The Oral-Based S2-Ag85A Vaccine Stimulates Humoral Immunity and Cytokine-Induced Responses in Mice

**Figure 4:**
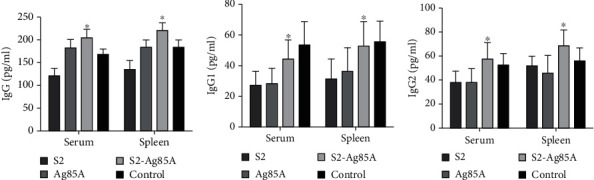
The S2-Ag85A vaccine induces immune responses. (a) In the serum and spleen of mice, the levels of IgG expression are significantly elevated in the S2-Ag85A group than that in the S2 group (*P* value < 0.05). (b) In the serum and spleen of mice, the levels of IgG1 expression are significantly elevated in the S2-Ag85A group than that in the S2 group (*P* value < 0.05). (c) In the serum and spleen of mice, the levels of IgG2a expression are significantly elevated in the S2-Ag85A group than that in the S2 group (*P* value < 0.05). ^∗^*P* < 0.05 compared with the S2 group.

Eight-week-old mice were injected with S2-ag85a oral vaccine and immunized three times. One week later, the mice were sacrificed, and the serum and spleen were collected to detect ELISA-based immune-specific antibodies with ELISA. In comparison with the control group, we found an elevated level of IgG secretion in the serum and spleen in the S2, Ag85A, and S2-Ag85A vaccinated groups (*P* value < 0.05). Among them, the IgG titers of mice could reach a range between 1 : 25000 and 1 : 40000 in the S2 and S2-Ag85A vaccinated groups. Furthermore, we analyzed the effect of the oral-based S2-Ag85A vaccine on IgG subtypes, and the ratio of Th2-related IgG1 and Th1-related IgG2a in the serum of each group was detected by using the ELISA. In comparison with the control group, the levels of IgG1 and IgG2a in the spleen and serum were significantly elevated in the S2, Ag85A, and S2-Ag85A vaccinated groups, and the ratio was higher in the spleen than in the serum. The ratios of Th1 and Th2 in serum were 0.46, 0.52, and 0.63; the ratios in the spleen were 0.65, 0.78, and 0.94, further indicating that the oral-based S2-Ag85A oral vaccine stimulates more significant Th1 responses (Figure 4), which suggested that the Th1 responses in serum and spleen were much more significant.

**Figure 5:**
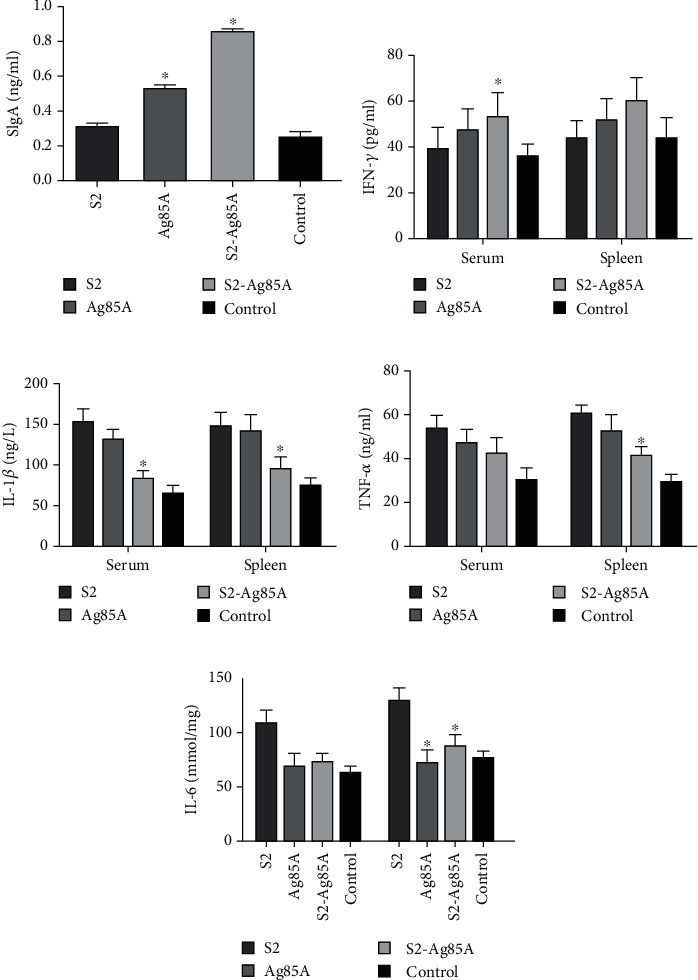
Enhancement of the cytokines stimulation by S2-Ag85A oral vaccine in mice. (a) The significantly elevated level of SIgA secreted by S2-Ag85A in the intestinal tract of mice than that in the S2 group (*P* value < 0.05). (b) The secretion of SIgA is augmented in peripheral serum and spleen tissue of mice after the application of S2-Ag85A vaccine. (c) The level of IL-1*β* is inhibited in peripheral serum and spleen tissue of mice after the application of S2-Ag85A vaccine (*P* value < 0.05). (d) The level of TNF-*α* is inhibited in the mouse spleen tissue after the application of S2-Ag85A vaccine (*P* < 0.05). (e) The level of IL-6 is inhibited in the mouse spleen tissue after the application of S2-Ag85A vaccine (*P* < 0.05). ^∗^*P* value < 0.05 compared with the S2 group.

We used the ELISA for detecting the SIgA titers in the small intestine of each group of mice. Mice from the S2-Ag85A and Ag85A groups could produce a mucosal IgA response after oral vaccination, but mice in the S2 group could not produce this response (Figure 5(a)).

To examine the cytokines stimulation potency of the oral-based S2-Ag85A vaccine, we examined the levels of crucial cytokines, including interferon-gamma (IFN-*γ*), interleukin (IL)-1*β*, tumor necrosis factor-alpha (TNF-*α*), and IL-6. Compared with mice in the blank group, the expression of IFN-*γ* was significantly increased in the peripheral serum and spleen of mice in the S2, Ag85A, and S2-Ag85A orally vaccinated groups (*P* value < 0.05). We found a significantly decreased level of IL-1*β*, TNF-*α*, and IL-6 expressions in the Ag85A group and S2-Ag85A orally vaccinated groups than that in the S2 group (*P* value < 0.05).

### 2.5. S2-Ag85A Oral Immunization Provides Better Immune Protection for Brucella Infection in Mice

**Figure 6:**
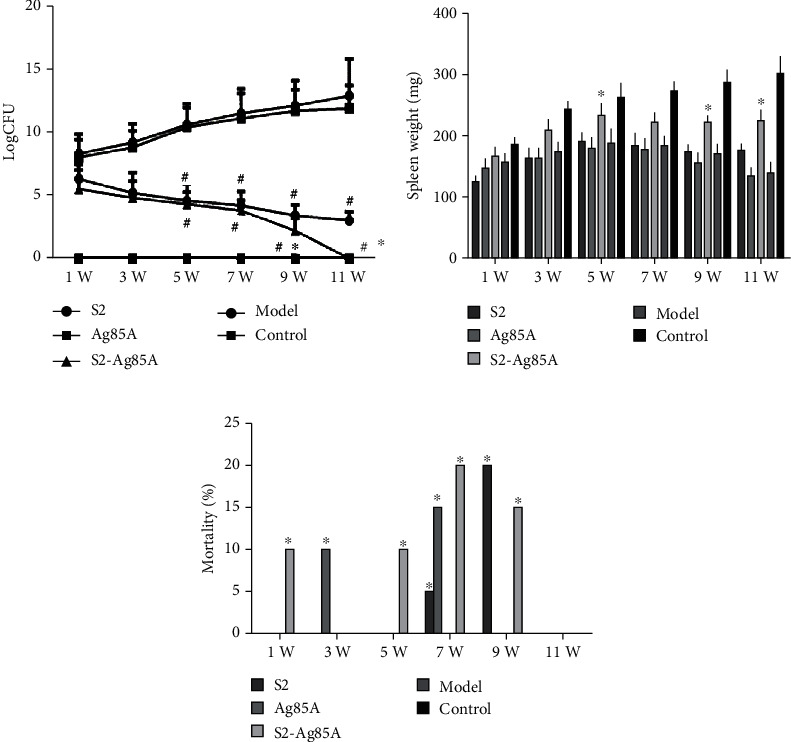
The S2-Ag85A oral vaccine provides better protection against Brucella infection in mice. (a) The bacterial content in the spleen of rats in each group was roughly the same one week after infection, and the bacterial content showed a significantly lower level in the spleen of mice in the S2 and S2-Ag85A groups than that in the model group at the 5th week (*P* < 0.05). (b) At each of 1-5 weeks, the weight of the spleen of mice in the group showed an upward trend. After 5 weeks, the spleen began to atrophy, and the weight of the spleen began to decrease. The mice in the 5, 9, and 11 W S2-Ag85A groups showed a significantly elevated weight of spleen than that in the model group (*P* value < 0.05). (c) From the first week, in the beginning, the S2 group, Ag85A group, and model group showed different degrees of death, and these group showed a significantly elevated death rate than that in the S2-Ag85A group (*P* value < 0.05).

To test the outcome of the oral-based S2-Ag85A vaccine on Brucella, we inoculated mice with 1 × 106 S2308 Brucella on the 7th day, after the three-session vaccination was completed. Spleen's weight and the number of Brucella in the spleen were assessed at 9 and 11 weeks. From week 1 to week 11, compared with mice in the control group, the burden of Brucella was significantly reduced in the spleen of mice in S2 and S2-Ag85A groups (*P* value < 0.05 Figure 6(a)); at week 11, S2, the spleen of mice in the Ag85A oral vaccine group was completely cleared of Brucella. According to the weight of the spleen, the weight of the spleen in the growth and development stage of the mice from 1 week to 5 weeks showed an upward trend. By comparing the model group of the treatment with the placebo, we found an elevated level of the spleen weight of mice in the S2-Ag85A oral vaccine group (*P* value < 0.05). The increase in the spleen weight of mice in the group was relatively low. From the 6th week to the 11th week, with the increase in the amount of virus replication, the spleen of mice in the model group, Ag85A, and S2 groups began to gradually shrink, and the spleen weight gradually decreased; this weight was significantly lower than that in the S2-Ag85A oral vaccine group (*P* value < 0.05 Figure 6(b)). After the injection of Brucella, we analyzed the mortality of mice. The model group, S2 group, and Ag85A group showed different numbers of mouse death from the 1st week to the 9th week, but the S2-Ag85A oral vaccine group did not show any mouse death. No mouse death occurred, and the mortality rate was significantly reduced (P < 0.05, Figure 6(c)).

## 3. Discussion

Although Brucella vaccines have been widely used, they still have various shortcomings, such as residual virulence, splenomegaly, or abortion in pregnant animals. The development of a vaccine with high protection rate and safety is an important challenge for scientists. The ideal Brucella vaccine should have a high protection rate and no residual toxicity, and it should effectively stimulate the immune responses of the body at the beginning of the vaccination. In this study, the S2-Ag85A oral vaccine was developed and evaluated; the toxicity and defensive ability in macrophages of mice were for a better understanding of its protective effect.

In this study, we proved the safety of the S2-Ag85A oral vaccine while verifying its effectiveness. After inoculating mice with Brucella, we found that it can effectively reduce the risk of infection in mice. In addition, we also found that the oral-based S2-Ag85A vaccine dominantly enhanced humoral immunity and induced Th1 cell responses.

To further examine the residual toxicity of the developed oral-based S2-Ag85A vaccine and the protecting capacity after the challenge with Brucella, we performed residual toxicity and protective assays in BALB/c mice. After inoculation of the S2-Ag85A oral vaccine in mice, the virus content in the spleen of mice gradually decreased from the first week to the 11th week; by the 10th week, the virus content in the spleen of mice completely disappeared. At the 9th and 11th weeks, the level of virus in the spleen of mice in the S2-Ag85A oral vaccine group was significantly higher than that in the S2 group (*P* value < 0.05). Besides, there was no obvious discomfort to mice during the vaccination period, which proved that oral administration of the S2-Ag85A vaccine can better avoid the problem of S2 vaccine toxicity residue. The protective test found that the S2-Ag85A oral vaccine had a good protective effect on the challenge of Brucella S2308. From the first week to the 11th week after being infected with the virus strain, the mortality rate in the S2 vaccine group was 5% and 20% in the 7th week and 9th week, respectively, and no death event occurred in the S2-Ag85A oral vaccine group, which proved that the protection rate of the S2-Ag85A oral vaccine was better than that of the S2 vaccine. Zhang [16] et al. used Ag85A as a vector to recombine the Mycobacterium tuberculosis VSV-846 vaccine and found that it effectively improved the immunogenicity of mice while enhancing the protection rate.

Ag85A is an extracellular secretory antigen. It was found that Ag85A promoted the proliferation of CD8+ T cells in mice, then induced augmentation of IFN-*γ* and TNF levels [17–19], and resisted the invasion of pathogenic microorganisms. Furthermore, we explored the immunogenicity of the oral-based S2-Ag85A vaccine in the body, we detected the immune indexes of body fluids and spleen in mice. The results showed that S2-Ag85A is associated with the secretion of IgG and IFN-*γ* in the blood and spleen of mice. Several studies indicated that macrophages are the key cells for sterilization, and the cytokine IFN-*γ* plays a major role. Studies have shown that when Ag85A is combined with Mtb32, it has a good protective effect on allergic asthma [20]. In the study, it was found that Ad5-gsgAM can induce more TH1 responses in mice. To further explore the TH1/TH2 immune response by orally administering the S2-Ag85A vaccine, we used the ELISA for detecting the IgG1 and IgG2 expression in the blood and spleen of mice. We found that the secretory level of IgG1 and IgG2 was significantly elevated in the S2-Ag85A group than that in the S2 vaccine, and the ratio of Th1 and Th2 was calculated to show that the S2-Ag85A vaccine induced a higher level of TH1 levels. Xu [21] et al. constructed the fusion expression protein Ag85A-IL-17A, and they found that it has a crucial protective role on allergic asthma in mice by augmenting the populations of TH1, inducing the production of autoimmunity, and slowing the secretion and expression of inflammatory factors. The Ag85A oral vaccine can effectively induce the production of antigen-specific mucosal cells and humoral immunity [22], and it can effectively induce the expression of sIgA in the intestine. In this study, the sIgA titer was detected in the intestinal tract of mice, and it was found that the sIgA level was increased after the S2-Ag85A oral vaccine, which is consistent with the characteristics of Ag85A.

Our findings showed that the oral-based S2-Ag85A vaccine is a powerful substitute for the original Brucella vaccine. The S2-Ag85A oral vaccine does not cause any toxicity in RAW 264.7 macrophages and mice. The S2-Ag85A oral vaccine has a better protective effect against Brucella S2308 infection. The S2-Ag85A oral vaccine can cause a specific IgG response of Brucella, and it can induce activation of the Th1 immune mechanism in mice. In future studies, we will further investigate the specific action pathway of the S2-Ag85A oral vaccine to protect the body and perform further studies in ruminants to test whether the S2-Ag85A oral vaccine is an effective alternative to the commonly used Brucella vaccine in China.

## 4. Materials and Methods

### 4.1. Mice

Briefly, we utilized 7-8-week-old BALB/C mice, including 18 males and 18 females that were purchased from Beijing Huafukang Biotechnology Co., Ltd. The license number for this experimental setup is SCXK (Beijing) 2019-0008 which was investigated and permitted by the Research Ethics Committee of Inner Mongolia University.

### 4.2. Vaccines

We purchased the S2 vaccine from Qilu Animal Health Co., Ltd. with approval number: (2015) 150257011. In addition, we developed the S2-Ag85A vaccine independently in our laboratory.

#### 4.2.1. Manufacturing the S2-Ag85A Vaccine

*(1) Qualified Manufacture.* We incubated Brucella (OD600 = 0.20) in TSA liquid medium for 15 minutes at 4°C and collected the bacteria after centrifugation at 4000 rpm for 5 minutes. The bacteria were washed two times by using deionized water and another time by using 15% glycerol. Each tube was filled with 100 *μ*L and stored at -80°C for later use.

*(2) CRISPR/CAS9 Plasmid Construction.* The target sequence of the S2 genome was determined, DNA oligonucleotide sequences of 20 bases corresponding to the 5 sgRNAs in the target sequence were designed and synthesized, and they were cloned into the CRISPR/Cas9 plasmid formed by the PX330 plasmid on the CAS9 plasmid. Five CRISPR/Cas9 plasmids were tested for activity, and we selected the most active CRISPR/Cas9 plasmid to cotransform the cells that showed sensitivity. The criteria of electric steering (no point transfer) were as follows: pulse voltage 1000 V, pulse width 40 ms, and no. 1 pulse 3. The target particle PTG2.0-Ag85a construction: we cloned the cDNA of Ag85a within the PTG2.0 vector and screened the resistant capacity of puromycin.

*(3) Detection of Positive Clone.* We utilized PCR (Ag85A-F: AAGTGGGAGACCTTCCTGACC; Ag85A-R: GAAGAAGCAGCCATCGAAAGA) and double digestion (Afl II and EcoR I sites) for determining the insertion of S2 gene into the Ag85A gene.

### 4.3. The Effect of the S2-Ag85A Oral Vaccine on Macrophages

We compared the survival rate of RAW264.7 macrophages after the application of the S2 vaccine and S2-Ag85A vaccine. In brief, 5^∗^105 cells/well were inoculated in a 24-well plate and incubated at 37°C for 24 hours. When the cell fusion rate was 60-70%, 25 *μ*g/ml S2/S2-Ag85A/Ag85A oral vaccine DMEM medium was added to the medium. At 4, 12, 24, and 48 hours after infection, we counted the viable bacteria by using the TSA plates. We completed all experiments at least three times.

### 4.4. Safety Evaluation of the S2-Ag85A Oral Vaccine in Mice

The toxicity of the S2-Ag85A oral vaccine was evaluated using BALB/C mice. Briefly, 8-week-old mice were administered 6^∗^104/0.1 ml of the S2-Ag85A oral vaccine and immunized three times; the first two immunizations were 10 days apart, and the second and third immunizations were 14 days apart. The S2 vaccine and Ag85A vector were administered by gavage in the same manner. We used a similar volume of normal saline for feeding the normal mice group. Mice were euthanized at 1, 3, 5, 7, 9, and 11 weeks after inoculation, and the spleen was aseptically collected and assayed for bacterial load; we lysed the splenocytes for making diluted suspension in sterile saline and spreader on the TSA medium. The bacterial CFU was counted after three days of incubation at 37°C. We repeated the experiment in triplicates.

### 4.5. Protective Capability of the Oral-Based S2-Ag85A Vaccine on Mice

We immunized the mice by inoculating the vaccine, and the process was the same as safety identification. Four weeks after the inoculation, mice of the S2 group, Ag85A group, model group, and oral-based S2-Ag85A vaccine group were inoculated with the 1 × 106 CFU/0.1 ml S2308 strain per mouse. The mice were euthanized by neck drag, 1-4 weeks after the challenge, and we further determined the Brucella content in the spleen and calculated the count of bacteria in the spleen. We repeated the experiment twice.

### 4.6. Antibody-Activated Immune Assessment

We collected the serum from the immunized mice at 1, 3, 5, 7, 9, and 11 weeks after immunization and evaluated the levels of IgG secretion by using the ELISA. We diluted the serum in PBS (1 : 100), and further prepared the two-fold serial dilutions. We used the ELISA Quantikine Mouse kit (R&D Systems, USA) for quantifying the IgG secretion in the prepared supernatant.

### 4.7. Detection of Cytokines

After eleven weeks of immunization, the spleen was removed under aseptic conditions after the blob was bled, and the mice were sacrificed. The protein content in the spleen was determined after the spleen was damaged by homogenization, and we quantified the expression level of cytokines including IgG, IFN-*α*, IL-1*β*, and TNF-*α* in the blood and spleen tissues.

## Figures and Tables

**Table 1:** Detection of hemolytic activity.

	S2	Ag85A	S2-Ag85A
0.25 h	—	—	—
0.5 h	+	—	—
0.75 h	+	—	—
1 h	++	—	—
2 h	++	—	—

## Data Availability

The data used to support this study is available from the corresponding authors upon request.
